# Routine Adoption of Urinary [IGFBP7]∙[TIMP-2] to Assess Acute Kidney Injury at Any Stage 12 hours After Intensive Care Unit Admission: a Prospective Cohort Study

**DOI:** 10.1038/s41598-019-52790-6

**Published:** 2019-11-11

**Authors:** Fiorenza Ferrari, Gregorio Romero-González, Lilia Rizo Topete, Mara Senzolo, Anna Lorenzin, Faeq Husain-Syed, Mariangela Valentina Puci, Ottavia Eleonora Ferraro, Eva Muraro, Mara Serrano-Soto, Alejandra Molano Triviño, Ana Coutinho Castro, Yun Xie, Bo Yang, Massimo De Cal, Valentina Corradi, Alessandra Brendolan, Marta Scarpa, Maria Rosa Carta, Davide Giavarina, Raffaele Bonato, Claudio Ronco

**Affiliations:** 1 International Renal Research Institute of Vicenza (IRRIV) and Department of Nephrology, Dialysis and Transplantation, San Bortolo Hospital, Viale Rodolfi, 37- 36100 Vicenza, Italy; 2 Intensive Care Unit, San Bortolo Hospital, Viale Rodolfi, 37- 36100 Vicenza, Italy; 3 Clínica Universidad de Navarra, Avenida Pio XII #36, 31008 Pamplona, Spain; 4 University Hospital “José Eleuterio González”, Francisco I Madero s/n and Gonzalitos, Colonia Mitras Centro - 64460 -Monterrey, Nuevo León, Mexico; 5 Department of Internal Medicine II, Division of Pulmonology, Nephrology and Critical Care Medicine, University Clinic Giessen and Marburg - Campus Giessen, 35392 Giessen, Germany; 6 Department of Public Health, Experimental and Forensic Medicine, Unit of Biostatistics and Clinical Epidemiology, University of Pavia, Via Forlanini, 2, 27100 Pavia, Italy; 7 DISCOG - Department of Surgery, Kidney and Pancreas Transplant, University of Padova, via Giustiniani 2, 35128 Padova, Italy; 8 Nephrology Service Nephrology Service Marques de Valdecilla – Universitary Hospital Valdecilla Avenue 25, 39008 Santander, Cantabria Spain; 9 Nephrology and Dialysis Service RTS - Fundación Cardioinfantil, calle 163 A N° 13B-60- 110111, Bogotá, Colombia; 10 Department of Nephrology, Dialysis and Transplantation. Oporto Hospital Center Largo Prof. Abel Salazar, 4099-001 Porto, Portugal; 11 Department of Nephrology, Xin Hua Hospital Affiliated to Shanghai Jiaotong University School of Medicine, South Chongqing Road no. 227, 200025 Shanghai, China; 12 Department of Nephrology, First Teaching Hospital of Tianjin University of Traditional Chinese Medicine, No. 88, Chang Ling Road, Li Qi Zhuang Jie, Xi Qing District, Tianjin, P.R. China; 13 Department of Laboratory Medicine, San Bortolo Hospital, Viale Rodolfi, 37- 36100 Vicenza, Italy

**Keywords:** Predictive markers, Kidney diseases, Acute kidney injury

## Abstract

The urinary tissue inhibitor of metalloproteinases-2 and insulin-like growth factor-binding protein 7 ([TIMP-2]∙[IGFBP7]) have been introduced to improve risk prediction of severe acute kidney injury (AKI) within 12 hours of measurement. We performed a prospective cohort study to evaluate if the predictive value of [TIMP-2]∙[IGFBP7] for AKI might continue after 12 hours. We enrolled 442 critically ill adult patients from June to December 2016. Urine samples were collected at admission for [TIMP-2]∙[IGFBP7] measurement. Baseline patient characteristics were recorded including patients’ demographics, prior health history, and the main reason for admission to build a logistic regression model to predict AKI. AKI occurrence differed between patients with [TIMP-2]∙[IGFBP7] ≤0.3 and >0.3 (ng/ml)^2^/1000 (31.9% and 68.10% respectively; p < 0.001). Patients with AKI had higher biomarker values compared to those without AKI (0.66 (0.21–2.84) vs 0.22 (0.08–0.63) (ng/ml)^2^/1000; p < 0.001). [TIMP-2]∙[IGFBP7] at ICU admission had a lower performance in predicting AKI at any stage within 48 hours and 7 days after measurement (area under the receiver operating characteristic curve (AUC) equal to 0.70 (95%CI 0.65–0.76), AUC 0.68 (95%CI 0.63–0.73)). In the logistic regression model, 0.1 (ng/ml)^2^/1000-unit increment was likely to increase the risk of AKI by 2% (p = 0.002).

## Introduction

Acute kidney injury (AKI) is estimated to occur in approximately 30–50% of patients admitted to the intensive care unit (ICU)^1,2^. AKI is a risk factor for future loss of kidney function, cardiovascular disease and death^3,4^. Increasing severity of AKI is associated with a prolonged ICU stay, renal replacement therapy requirement, the use of resources and death^5,6^.

According to the current definition of AKI, an acute worsening of renal function is linked to increased serum creatinine (SCr) and/or reduced urinary output. Due to SCr kinetics, however, AKI is diagnosed 48–72 hours after it has occurred, and factors such as hydration, nutrition, and lean tissue status further confound the diagnosis^7^. Several biomarkers have been identified for timely diagnosis of AKI and AKI risk stratification. These may allow us to detect “subclinical AKI”, a condition in which kidney injury may exist without fulfilling the current consensus criteria^8^ but potentially lead to overt clinical AKI^9,10^. The commercially available NephroCheck® Test is an immunoassay test that quantitatively measures the product of urinary concentrations of insulin-like tissue inhibitor of metalloproteinases-2 and growth factor-binding protein 7 ([TIMP-2]∙[IGFBP7]) and combines them into a single numerical result. These so-called cell cycle arrest biomarkers represent soluble proteins expressed and secreted by the proximal and distal renal tubular cells and have been identified as more sensitive than serum creatinine in detecting kidney injury or stress^11^.

[TIMP-2]∙[IGFBP7] was validated in the Sapphire Study, in which the authors highlighted its remarkable ability to predict the development of moderate and severe AKI (stage 2 to 3) within 12 hours of sample collection^12^. A biomarker cut-off of 0.3 (ng/ml)^2^/1000 was established to achieve high sensitivity while preserving acceptable specificity^12–14^. Patients with [TIMP-2]∙[IGFBP7] values > 0.3 (ng/ml)^2^/1000 have a seven-times higher risk of developing AKI (95% CI 4–22) compared to patients with lower levels^14^.

Since June 2016, [TIMP-2]∙[IGFBP7] has been introduced at our ICU in addition to traditional measures of AKI as part of routine clinical practice to evaluate its clinical utility. Our study shows the predictive value of these biomarkers at ICU admission for the development of AKI over time, proposing a model to predict the risk of AKI at any stage^7^.

## Results

### Acute kidney injury rates and biomarker performance

Four hundred forty-two patients were recruited at our multidisciplinary ICU from June 1 to December 31, 2016, of which 62.40% were male and the mean age was 63.69 ± 17.90 years.

The AKI occurrence within 7 days was 42.53% (n = 188), of which 125 (66.49%) patients were categorized as stage 1, 34 (18.09%) as stage 2, and 29 (15.43%) as stage 3 AKI (Supplementary Fig. 1S). Patients with AKI within 12 hours were around 58% (n = 116), 34% (n = 53) had AKI between 12 hours and 48 hours and 8% (n = 19) after 48 hours.

More prevalent admission diagnosis was trauma (20.60%), then sepsis (18.32%) (Table 1). Baseline and post-24-hour hospitalization characteristics are provided for all the sample and for each group ([TIMP-2]∙[IGFBP7] values > 0.3 (ng/ml)^2^/1000) or ≤0.3 (ng/ml)^2^/1000)) in Table 2.

**Table 1:** ICU Admission Diagnosis.

Diagnosis	n (%)
Surgery*	64 (14.48)
Trauma	97 (21.95)
Neurologic	52 (11.76)
Sepsis	80 (18.10)
Cardiac	44 (9.95)
Respiratory	54 (12.22)
Other	51 (11.54)
Total	442 (100)

*Urgent non-cardiac surgery.

**Table 2:** Characteristics of ICU Admission, 24 Hours Post-Admission and Renal Function in all patients with [TIMP-2]•[IGFBP7] ≤ 0.3 and [TIMP-2]•[IGFBP7] > 0.3 ((ng/ml)2/1000).

	All patients (n = 442)	TIMP-2]∙ [IGFBP7] ≤ 0.3 (n = 206)	TIMP-2]∙ [IGFBP7] > 0.3 (n = 236)	P-value
**Admission**				
Male, n (%)	276 (62.4)	126 (61.2)	150 (63.6)	0.604
Age (years)	68.00 (52.00–78.00)	67.00 (51.00–77.00)	69.00 (54.00–79.00)	0.140
Weight (kg)	75.00 (65.00–85.00)	75.00 (65.00–85.00)	75.00 (65.00–85.00)	0.320
Height (cm)	171.70 ± 7.70	172.13 ± 7.72	171.40 ± 7.767	0.330^§^
BMI (kg/m^2^)	24.80 (22.77–27.76)	24.69 (22.49–27.06)	25.14 (22.86–28.41)	0.087
Hypertension, n (%)	195 (44.1)	93 (45.1)	102 (43.2)	0.680
Diabetes mellitus type 2 (%)	76 (17.2)	35 (17.0)	41 (17.4)	0.900
eGFR (ml/min/1.73 m^2^)	83.00 (52.00–100.00)	90.00 (64.00–107.00)	73.00 (45.00–92.00)	<0.001
SAPS II	40.00 (29.00–51.00)	37.50 (27.00–50.00)	42.00 (31.00–52.00)	0.031
SOFA	6.00 (4.00–9.00)	6.00 (3.00–8.00)	7.00 (5.00–10.00)	<0.001
MAP (mmHg)	81.00 (69.00–98.00)	87.50 (71.00–101.00)	78.50 (67.00–95.00)	<0.001
PEEP (cmH_2_O)	6.00 (0.00–8.00)	6.00 (0.00–8.00)	6.00 (0.00–8.00)	0.430
PaO_2_/FiO_2_	295.00 (195.00–406.00)	310.00 (222.00–437.93)	276.00 (182.50–387.50)	0.024
pH	7.40 (7.33–7.45)	7.41 (7.34–7.46)	7.40 (7.32–7.45)	0.110
Lactate (mmol/l)	1.80 (1.30–2.90)	1.60 (1.20–2.30)	2.10 (1.50–3.60)	<0.001
PaCO_2_ (mmHg)	38.10 (33.70–44.00)	38.00 (34.00–44.00)	38.70 (33.35–44.15)	0.900
HCO_3_ (mmol/l)	23.70 (21.00–26.20)	23.70 (21.90–26.50)	23.40 (20.20–25.90)	0.160
BE (mmol/l)	−1.30 (−4.10–1.90)	−1.10 (−3.60–2.60)	−1.55 (−5.00–1.40)	0.110
Hb (g/dl)	11.70 ± 2.30	11.85 ± 2.21	11.60 ± 2.29	0.241^§,^*
PCT (μg/l)	0.69 (0.16–4.47)	0.36 (0.11–1.43)	1.32 (0.26–10.81)	<0.001
Nephrotoxic drugs, n (%)**	131 (29.8)	56 (27.2)	75 (32.1)	0.270
**24 hours**				
Diuresis (ml)	1767.50 (1155.00–2550.00)	1975.00 (1330.00–2815.00)	1637.50 (1000.00–2347.50)	0.001
Fluid balance (ml)	694.00 (−278.00–1653.90)	412.00 (−522.50–1326.00)	882.00 (−98.80–1955.00)	<0.001
Furosemide (mg)	0.00 (0.00–30.00)	0.00 (0.00–20.00)	0.00 (0.00–40.00)	0.084
Dopamine (μg/kg/min)	0.00 (0.00–0.00)	0.00 (0.00–0.00)	0.00 (0.00–0.00)	0.005
Dobutamine (μg/kg/min)	0.00 (0.00–0.00)	0.00 (0.00–0.00)	0.00 (0.00–0.00)	0.410
Epinephrine (μg/kg/min)	0.00 (0.00–0.00)	0.00 (0.00–0.00)	0.00 (0.00–0.00)	0.240
Norepinephrine (μg/kg/min)	0.00 (0.00–0.05)	0.00 (0.00–0.00)	0.00 (0.00–0.08)	<0.001
Terlipressin (UI/min)	0.00 (0.00–0.00)	0.00 (0.00–0.00)	0.00 (0.00–0.00)	0.003
MAP (mmHg)	80.60 ± 15.70	81.30 ± 15.36	80.03 ± 15.99	0.415^§^
PEEP (cmH_2_O)	6.00 (0.00–8.00)	6.00 (0.00–8.00)	7.00 (0.00–8.00)	0.260
PaO_2_/FiO_2_	292.00 (210.00–372.50)	303.50 (217.75–381.25)	282.00 (206.44–362.50)	0.210
pH	7.46 (7.41–7.50)	7.46 (7.42–7.50)	7.46 (7.41–7.50)	0.250
PaCO_2_ (mmHg)	38.00 (34.00–43.00)	38.00 (34.00–43.00)	38.95 (34.00–44.00)	0.380
HCO_3_ (mmol/l)	26.55 (24.00–29.10)	26.35 (24.50–28.30)	27.30 (23.20–29.90)	0.630
BE (mmol/l)	2.85 (0.25–5.00)	2.55 (0.50–4.75)	3.10 (−0.65–5.60)	0.990
Hb (g/dl)	10.85 (9.70–12.20)	10.75 (9.70–12.20)	10.95 (9.70–12.20)	0.550
Lactate (mmol/l)	1.40 (1.10–2.10)	1.30 (1.00–1.80)	1.60 (1.20–2.50)	<0.001
Transfusion, n (%)	100 (23.2)	46 (22.7)	54 (23.7)	0.800
**Nephrology**				
SCr baseline (mg/dl)	0.77 (0.60–0.95)	0.74 (0.59–0.94)	0.78(0.61–0.95)	0.420
eGFR baseline (ml/min/1.73 m^2^)	98.56 (76.24–132.81)	100.29 (77.73–134.63)	98.28 (75.41–129.56)	0.370
SCr at ICU admission (mg/dl)	0.91 (0.68–1.29)	0.80 (0.61–1.05)	0.96 (0.78–1.50)	<0.001
SCr at 24 h (mg/dl)	0.89 (0.69–1.35)	0.79 (0.66–1.11)	0.97 (0.74–1.62)	<0.001
SCr at 48 h (mg/dl)	0.88 (0.66–1.25)	0.81 (0.64–1.16)	0.93 (0.68–1.33)	0.065
SCr at 72 h (mg/dl)	0.82 (0.64–1.14)	0.79 (0.61–1.12)	0.87 (0.68–1.24)	0.110
SCr discharge (mg/dl)	0.81 (0.60–1.08)	0.74 (0.60–1.04)	0.86 (0.62–1.20)	0.031
CRRT, n (%)	20 (4.6)	4 (2.0)	16 (6.9)	0.014
[TIMP-2]∙[IGFBP7] ((ng/ml)2/1000)	0.36 (0.10–1.18)	0.09 (0.04–0.17)	1.10 (0.52–3.04)	<0.001

^§^Equal variance t-test, *unequal variance t-test.

Data are presented as median (25th-75th) or mean and standard deviation for continuous measures, and n (%) for categorical measures.

**Nephrotoxic drugs included aminoglycosides, non-steroidal anti-inflammatory drugs, and vancomycin.

BMI = body mass index; BE = base excess; RRT = renal replacement therapy; eGFR = estimated glomerular filtration rate; Hb = hemoglobin; IGFBP7 = insulin-like growth factor-binding protein 7; MAP = mean arterial pressure; PEEP = positive end-expiratory pressure; PaO_2_/FiO_2_ = ratio of the partial pressure of oxygen in arterial blood to inspired oxygen fraction; PCT = procalcitonin; SAPS II = simplified acute physiology score II; SCr = serum creatinine; SOFA = sequential organ failure assessment; TIMP-2 = tissue inhibitor of metalloproteinases 2.

Highest values of [TIMP-2]∙[IGFBP7] were found in patients underwent urgent non-cardiac surgery (Table 3). The association between diagnosis and the occurrence of AKI was borderline (χ^2^ = 12.80, p value = 0.050 data not shown).

**Table 3:** [TIMP-2]•[IGFBP7] value ((ng/ml)2/1000)) according to the ICU admission diagnosis.

Diagnosis	[TIMP-2]•[IGFBP7] median (25^th^–75^th^)
Surgery*	0.75 (0.24–3.38)
Trauma	0.20 (0.08–0.53)
Neurologic	0.39 (0.11–0.73)
Sepsis	0.21 (0.06–0.55)
Cardiac	0.41 (0.10–1.79)
Respiratory	0.70 (0.21–2.12)
Other	0.31 (0.09–2.54)

*Urgent non-cardiac surgery. IGFBP7 = insulin-like growth factor-binding protein 7; TIMP-2 = tissue inhibitor of metalloproteinases 2.

At admission, patients with [TIMP-2]∙[IGFBP7] values > 0.3 (ng/ml)^2^/1000) showed a more severe critical illness than patients with [TIMP-2]∙[IGFBP7] ≤ 0.3 (ng/ml)^2^/1000) as the Simplified Acute Physiology Score II (SAPS II) (p = 0.031), the Sequential Organ Failure Assessment (SOFA) (p < 0.001), lactate (p < 0.001) and Procalcitonin values (p < 0.001) describe (Table 2).

In AKI patients, biomarker values were higher compared to those who did not develop AKI (0.66 (0.21–2.84) ((ng/ml)^2^/1000) vs 0.22 (0.08–0.63) ((ng/ml)^2^/1000); p < 0.001). Furthermore, patients who developed AKI within 12 and 48 hours showed higher biomarker values compared to patients who developed AKI later (respectively, 1.39 (0.41–4.76) ((ng/ml)^2^/1000) and 0.43 (0.17–1.03) ((ng/ml)^2^/1000) versus 0.22 (0.07–0.52) ((ng/ml)^2^/1000); p < 0.001). AKI occurrence was higher in patients with [TIMP-2]∙[IGFBP7] >0.3 ((ng/ml)^2^/1000). More specifically, 31.90% in patients with [TIMP-2]∙[IGFBP7] ≤ 0.3 ((ng/ml)^2^/1000) and 68.10% in patients with >0.3 ((ng/ml)^2^/1000) (p < 0.001). AKI stage 1 occurrence was 80% and 60.16% in the patients with [TIMP-2]∙[IGFBP7] ≤ 0.3 ((ng/ml)^2^/1000) and [TIMP-2]∙[IGFBP7] > 0.3 ((ng/ml)^2^/1000), respectively. Otherwise, the prevalence of severe AKI (KDIGO stage 2 and 3) was higher in the patients with [TIMP-2]∙[IGFBP7] > 0.3 (ng/ml)^2^/1000) (p = 0.026) (Supplementary Table 1S).

### Predictive value of biomarkers for AKI

**Figure 1:**
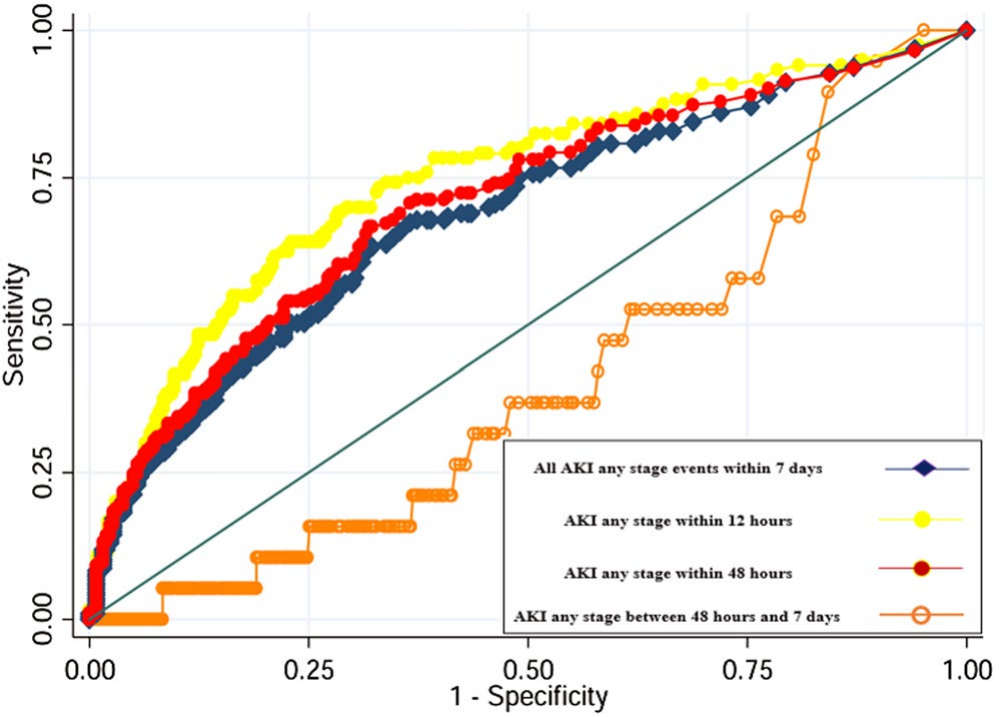
Area under the receiver-operating characteristics curve (AUC) for [TIMP-2]*[IGFBP7] to predict AKI any stage within 7 days, within 48 hours and AKI within 12 hours. *All AKI* (any stage or stage 2 and 3) *within 7 days* means all AKI events up to 7 days; *AKI within 48 hours* (any stage or stage 2 and 3) means events occurred from 12 hours to 48 hours.

**Figure 2:**
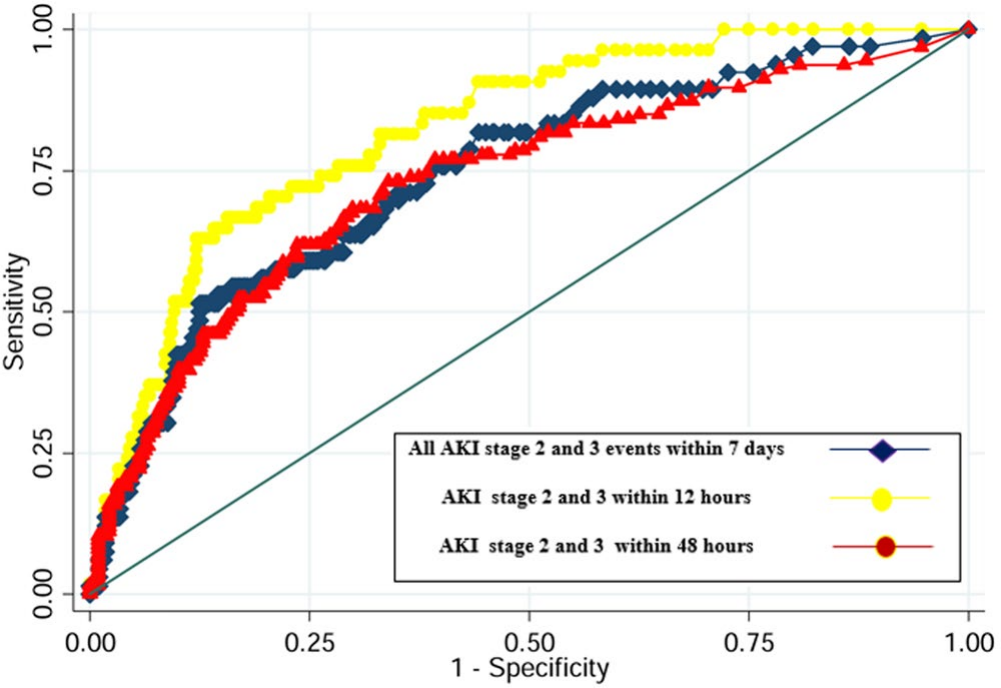
Area under the receiver-operating characteristics curve (AUC) for [TIMP-2]*[IGFBP7] to predict all AKI stage 2 and 3 events within 7 days of ICU admission, AKI stage 2 and 3 within 48 hours and AKI stages 2–3 within 12 hours. *All AKI* (any stage or stage 2 and 3) *within 7 days* means all AKI events up to 7 days; *AKI within 48 hours* (any stage or stage 2 and 3) means events occurred from 12 hours to 48 hours. Since few patients developed severe AKI after 48 hours, ROC curve for AKI stage 2 and 3 after 48 hours has not been performed.

We performed an ROC curve to evaluate the predictive ability of [TIMP-2]∙[IGFBP7] for AKI: patients with AKI within 12 hours (AUC 0.74 (95%CI 0.69–0.80)), 48 hours (AUC 0.70 (95%CI 0.65–0.76)) and between 48 hours and 7 days (AUC 0.40 (95%CI (95%CI 0.28–0.52)) (Figs 1 and 2; Supplementary Table 2S). [TIMP-2]*[IGFBP7] showed the best performance for severe AKI (stages 2 and 3) within 12 hours: AUC 0.82 (95%CI 0.70–0.88)) and all events of severe AKI up to 7 days (AUC 0.74 (95%CI 0.68–0.81); Figs 1 and 2, Supplementary Table 2S). Using the Liu method^15^, we identified significant differences in biomarkers cut-off to discriminate patients with AKI at different timepoints and severity. (Supplementary Table 3S). All empirical optimal cut points were higher than 0.3 ((ng/ml)^2^/1000)^13^ and showed a better specificity. The entire list of sensitivity and specificity can be found as Supplementary Tables 3S and 4S.

Table 4 shows results of the multivariable logistic regression using [TIMP-2]*[IGFBP7] as a continuous variable. Otherwise, Table 5 shows results of the multivariable logistic regression with or without [TIMP-2]*[IGFBP7] as a dichotomous variable.

**Table 4:** Multivariable Logistic Regression Analysis of Factors Associated with AKI Within 7 days of ICU Admission and [TIMP-2]∙[IGFBP7]considered as a continuous variable.

Parameters	All patients	
	OR (95% CI)	p-value
BMI	1.01 (0.97–1.06)	0.573
eGFR at admission	0.98 (0.98–0.99)	<0.001
SOFA	1.11 (1.03–1.19)	0.007
PEEP	1.02 (0.96–1.09)	0.489
Lactate	1.00(0.92–1.10)	0.946
[TIMP-2]•[IGFBP7]	1.21 (1.07–1.36)	0.002

Area under the receiver operating characteristic curve (95% CI) = 0.75 (0.69–0.79); goodness of fit p = 0.35; Se = 56.49, Sp = 82.23. IDI Estimate = 0.031 standard error = 0.009 p = 0.001. [TIMP-2]•[IGFBP7] is considered as a continuous variable.

**Table 5:** Multivariable Logistic Regression Analysis of Factors Associated with AKI within 7 days of ICU Admission: the first without [TIMP-2]∙[IGFBP7] and the second with the added of [TIMP-2]∙[IGFBP7].

Parameters	OR (95% CI)*	p-value	OR (95% CI)**	p-value
BMI	1.02 (0.97–1.07)	0.396	1.02 (0.97–1.07)	0.453
eGFR admission	0.98 (0.97–0.99)	<0.001	0.98(0.97–0.99)	<0.001
SOFA	1.10 (1.02–1.18)	0.012	1.09 (1.01–1.17)	0.022
PEEP	1.01 (0.95–1.08)	0.656	1.02 (0.96–1.08)	0.568
Lactate	1.05 (0.96–1.14)	0.294	1.03 (0.94–1.12)	0.548
[TIMP-2]•[IGFBP7] > 0.3	—	—	2.08 (1.29–3.37)	0.003

*Area under the receiver operating characteristic curve (95% CI) = 0.75 (0.71–0.79); goodness of fit p = 0.27; Se = 59.09, Sp = 79.19. Model without [TIMP-2]•[IGFBP7].

**Area under the receiver operating characteristic curve (95% CI) = 0.75 (0.71–0.79); goodness of fit p = 0.32; Se = 59.09, Sp = 79.70. IDI Estimate = 0.022 standard error = 0.008 p = 0.005.

[TIMP-2]•[IGFBP7] is considered as a dichotomous variable.

AKI = acute kidney injury; BMI = body mass index; eGFR = estimated glomerular filtration rate; ICU = intensive care unit; IGFBP7 = insulin-like growth factor-binding protein 7; PEEP = positive end-expiratory pressure; SOFA = sequential organ failure assessment; TIMP-2 = tissue inhibitor of metalloproteinases 2.

In the multivariable logistic regression model, the relevant factors associated with an increased risk of AKI were SOFA (OR 1.11; 95% CI 1.03–1.19; p = 0.007) and [TIMP-2]∙[IGFBP7] measurement: a 0.1 (ng/ml)^2^/1000 unit increment was likely to increase the risk of AKI by 2% (p = 0.002, Table 4). On the other hand, eGFR represents a protective factor: there was a moderate decrease in the risk of AKI (OR 0.98; 95% CI 0.97–0.98; p < 0.001).

Patients with biomarker values > 0.3 ((ng/ml)^2^/1000) had a two-fold risk of developing AKI (OR 2.08; 95% CI 1.29–3.37; p = 0.003; Table 5).

Finally, the clinical parameters associated with the need for CRRT at ICU admission were eGFR (OR 0.95; 95% CI 0.92–0.98; p < 0.001) and SOFA (OR 1.26; 95% CI 1.05–1.51; p = 0.015), but not [TIMP-2]∙[IGFBP7] (OR 1.88; 95%CI 0.46–7.66; p = 0.377) (Table 6).

**Table 6:** Regression Analysis of Risk Factors Associated with CRRT in ICU.

Parameters	OR (95% CI)	P-value
BMI	1.05 (0.96–1.16)	0.274
eGFR at admission	0.95 (0.92–0.98)	<0.001
SOFA	1.26 (1.05–1.51)	0.015
PEEP	0.99 (0.85–1.16)	0.913
Lactate	1.09 (0.97–1.22)	0.136
[TIMP-2]•[IGFBP7] > 0.3	1.88 (0.46–7.66)	0.377

BMI = body mass index; CCA = cell cycle arrest; eGFR = estimated glomerular filtration rate; ICU = intensive care unit; IGFBP7 = insulin-like growth factor-binding protein 7; PEEP = positive end-expiratory pressure; SOFA = sequential organ failure assessment; TIMP-2 = tissue inhibitor of metalloproteinases 2.

## Discussion

AKI is considered as a complex clinical syndrome, and it has recently been revisited as a continuum from increased susceptibility to irreversible kidney failure. New biomarkers may allow us to detect “subclinical AKI”, a condition in which kidney injury may exist without fulfilling the current consensus criteria^8^ but potentially lead to clinical AKI^9,10^. No specific therapy exists for AKI: KDIGO suggests a supportive management to prevent AKI, limiting or avoiding a possible its progression^7^. In this context, early AKI diagnosis might be an efficient method to carry out different strategies during a short “therapeutic window” in order to avoid the renal function worsening^16^.

In 2016, we introduced [TIMP-2]∙[IGFBP7] into our routine clinical practice; in this observational cohort study we investigated the utility of [TIMP-2]∙[IGFBP7] measurement at ICU admission for the early identification of patients at risk of AKI. In our study, AKI occurrence was 42.53% (n = 188) within 7 days, with 89.89% (n = 169) of these patients developing AKI within 48 hours. In patients who developed AKI stage 1, the median value of [TIMP-2]∙[IGFBP7] was 0.50 (0.16–1.69) (ng/ml)^2^/1000. This value is higher than the well-known cut-off that predicts severe AKI. Accordingly, the recently published AUC for developing severe AKI^12–14^ and the respective cut-off point for [TIMP-2]∙[IGFBP7] could not be confirmed in our cohort: we found a lower AUC that only improved when AKI within 48 hours and severe AKI were considered. In addition, we found a higher biomarker cut-off point to identify AKI patients. The opposite of previous studies^12–14^, [TIMP-2]∙[IGFBP7] showed exclusively a better prediction ability to detect no AKI patients. Furthermore, in our population the cut-off proposed by Hoste EAJ *et al*.^13^ maintains a good sensitivity, but it fails to identify the true negative patients.

The reason for these findings may lie in different patient cohort compositions as well as in study design and endpoints: our study explores the predictive ability of [TIMP-2]∙[IGFBP7] for AKI at any stage within 7 days in a multidisciplinary ICU population. Additionally, more than half of the AKI cohort (66.49%) developed AKI stage I.

A previous study demonstrated an improvement in the performance of some biomarkers in diagnosing AKI renal damage for up to 12 hours: neutrophil-gelatinase-associated lipocalin (NGAL) obtained the best AUC between 12 and 36 hours, while ɣ-glutamyl transpeptidase (GGT) predicted AKI soon after insult (6–12 hours)^17^

The differences between biomarkers reflect the time course of each biomarker: a pre-formed enzyme (GGT) appears soon in the urine and is rapidly depleted; on the other hand, the expression of other biomarkers is induced by a kidney injury (NGAL, [TIMP-2]∙[IGFBP7]) or presumably reflects increasing proximal tubular injury and impaired reabsorption (Cystatin C).

Furthermore, few studies have not focused on predicting severe AKI (stage 2–3). Among these, surgical patients were the target population, including patients undergoing coronary artery bypass graft surgery with cardiopulmonary bypass^18^, valvular, or combined surgery with cardiopulmonary bypass^19^ and no cardiac surgery^20^. Meersch^21^ and Gocze^22^ estimated an AUC for AKI stage 1 of 0.81 and 0.85, respectively. With an estimated cut-off of 1.1 (ng/ml)^2^/1000, Wetz and colleagues were able to discriminate between patients with and without AKI on the first postoperative day with an AUC of 0.706, albeit this study did not include high risk patients and those undergoing urgent surgery^18^. Otherwise, Di Leo *et al*. evaluated the value of [TIMP-2]∙[IGFBP7] to predict AKI free days in 719 critically ill patients, estimating an optimal cut-off of 0.37 (ng/ml)^2^/1000 (AUC 0.633, specificity and sensitivity 56% and 64%, respectively), thereby demonstrating that [TIMP-2]∙[IGFBP7] captures most AKI positive cases and a high number of patients who do not develop AKI^21^.

AKI diagnosed from stage 1 criteria is affected by many factors involving haemoconcentration, drugs, and reversible oliguria that may not develop into real kidney stress and damage. Although KDIGO stage 2 or 3 reflects a moderate-to-severe AKI, renal cells are more likely to sustain insults by sepsis and ischemia that cause kidney damage^8,22^. Based on this hypothesis, urinary [TIMP-2]∙[IGFBP7] may be more sensitive in predicting severe AKI as opposed to AKI stage 1^22,23^. In fact, also in our study [TIMP-2]∙[IGFBP7] showed the best performance to predict severe AKI by 12 hours and 48 hours. Although a smaller occurrence of late AKI in our study, [TIMP-2]∙[IGFBP7] might have a low performance to predict AKI after 48 hours (AUC 0.40) per se, as previously shown for NGAL and GGT^17^.

Our ICU population is heterogeneous, having different admission diagnoses and different degrees of severity in critical illness, as the 25^th^ and 75^th^ percentiles of SAPSII and SOFA scores proved in our study. While in cardiac or major surgery the timing of kidney injury is known, patients who are admitted to the ICU may be at any point of the hypothetical “AKI timeline”, which may range from exposure to renal injury to irreversible kidney damage. Furthermore, during critical illness, patients are exposed to a large number of insults, often simultaneously and/or in succession, that are potentially harmful to renal function. In this context, a unique [TIMP-2]∙[IGFBP7] measurement may not able to mirror the complex dynamic of kidney injury, because this accuracy might progressively decrease over time.

To address this issue, we built a multivariable logistic regression model on the cohort to identify which clinical covariates at admission may affect AKI occurrence. Consequently, the best model to predict AKI development up to 7 days included the results of the [TIMP-2]∙[IGFBP7] as a continuous or categorical covariate.

As far as the [TIMP-2]∙[IGFBP7] value is concerned, a rise of 0.1 (ng/ml) 2/1000 increased the risk of AKI of 2% (OR 1.21; 95% CI 1.07–1.36; p = 0.002) (Table 4). Furthermore, in patients who had biomarker values > 0.3 (ng/ml) 2/1000, the risk of AKI at any stage was double that of patients who had values ≤ 0.3 (ng/ml) 2/1000 (OR 2.08; 95% CI 1.27–3.42; p = 0.004).

For this reason, we estimate that a combined approach and a different use of [TIMP-2]∙[IGFBP7] could represent the most successful diagnostic strategy for predicting AKI also up to 7 days: combining a clinical risk model with the [TIMP-2]∙[IGFBP7] trend correlated to the patient’s status. However, it may be more appropriate to identify a subset of patients in whom [TIMP-2]∙[IGFBP7] will be utilised more efficiently, evaluating the trend during the first days of their ICU stay. In fact, the timing of the test performance would deserve careful consideration: on the one hand, and referring to an early AKI diagnosis, if the test is run too early, biomarkers might not have increased sufficiently to detect the risk of kidney injury. On the other hand, the admission [TIMP-2]∙[IGFBP7] measurement might simply be the rise or fall of the biomarker kinetic curve^24^.

The routine adoption of [TIMP-2]∙[IGFBP7] combined with clinical parameters commonly used in the ICU could represent a proposal for detecting the risk of developing AKI at admission^25^. Like procalcitonin in sepsis, the role of [TIMP-2]∙[IGFBP7] is to guide the clinician in a timely manner for further intervention of the patients at risk. While the time window for intervention or the algorithm of therapy have been well identified for other acute conditions with high morbidity and mortality, no such timeframe or specific therapy has been definitively clarified for AKI, even if some previous studies have identified a time window for intervention for AKI^26,27^. The UK National Health Service found that delayed identification of patients with AKI contributes to worsening outcomes^28^. Our model proposes a warning to the physician upon a patient’s ICU admission, leading to a substantial shortening of the time needed for the intervention (at admission to the ICU and at any stage), suggesting that when [TIMP-2]∙[IGFBP7] is greater than 0.3, an increased risk of AKI may persist up to 7 days.

Finally, in our study [TIMP-2]∙[IGFBP7] measurement at admission failed to predict CRRT in critically ill patients, probably due to the low occurrence of RRT. Of note, eight patients received CRRT within 10 days of ICU admission to manage fluid overload, although they did not develop AKI. Five of the patients exhibited [TIMP-2]∙[IGFBP7] values > 0.3 (ng/ml)^2^/1000.

This study has some limitations. First, it is a single-centre investigation in which the study design allowed only an exploratory analysis. Moreover, intensivists were not blinded to the [TIMP-2]∙[IGFBP7] results. Despite these limitations, we believe that our data acknowledges the diagnostic utility of [TIMP-2]∙[IGFBP7], which could offer an adjunctive tool in order to avoid AKI progression and perform different actions plan to enable the intensivist prioritise their clinical decision-making in the context of AKI any stage risk assessment. Additional randomised studies are required in a stratified population by adopting a mixed risk score (biomarker test results and clinical parameters) to gain more information on how major renal adverse events can be predicted and therefore improve the survival rate of AKI patients.

## Conclusion

In patients admitted to a multidisciplinary ICU, the admission measurement of urinary [TIMP-2]∙[IGFBP7] has a lower performance in the risk assessment of AKI over time compared to its use in detecting AKI within 24 hours. The best performance of the unique measurement of [TIMP-2]∙[IGFBP7] at the ICU admission remains for severe AKI. The initial measurement of urinary [TIMP-2]∙[IGFBP7] might be optimised by inserting it in a clinical score or using its trend. Further studies are warranted to optimise the use of this biomarker, focusing on its timing and trend.

## Methods

### Setting and study population

This observational prospective cohort single-centre study was conducted in the multidisciplinary ICU at San Bortolo Hospital, Vicenza, Italy. Study approval was obtained from the local Human Research Ethics Committee; the study complied with the Declaration of Helsinki. The informed consent to participate was obtained pursuant to Italian laws.

The study design, execution and report meet the STROBE criteria (STrengthening the Reporting of OBservational Studies in Epidemiology, also known as STROBE)^29^. Data was collected by the investigators and analyzed by external statisticians.

From June 1 to December 31, 2016, a total of 450 consecutive patients were admitted to our ICU. The inclusion criteria were: age ≥18 years and urinary catheter in place for at least 48 hours, while exclusion criteria were: age <18 years, chronic kidney disease (CKD), defined as kidney damage for ≥3 months, as defined by structural or functional abnormalities of the kidney, with or without decreased GFR or GFR^30^, patients with anuria or with diuresis less than 30 ml within 24 hours of ICU admission, and a presumed life expectancy of less than 48 hours after admission (Fig. 2).

### Definition and staging of acute kidney injury and assessment of baseline serum creatinine

The diagnosis and staging of AKI were determined based on serum creatinine (SCr) and urine output (UO) using the criteria in Kidney Disease: Improving Global Outcomes (KDIGO)^7^. To assess the baseline creatinine, we used the pre-morbid SCr measured 90–180 days before ICU admission when available^31^. In those patients where pre-morbid SCr was not available, two different strategies were adopted: (a) we used the lowest in-patient SCr assessed during the first 10 days of ICU admission when creatinine was <1.5 mg/dL and without dialysis requirement, as this scenario falls under the category of renal recovery^32,33^; (b) we used the back-calculation formula:

1 $$({\rm{Baseline}}\,{\rm{sCr}}=(75/(186\times ({\rm{age}}-0.203)\times (0.742\,{\rm{if}}\,{\rm{female}}))-0.887)$$

Ref.^34^ for patients with SCr ≥ 1.5 mg/dL.

### Data collection

Baseline patient characteristics were recorded, including patients’ demographics, prior health history, and the main reason for admission. The severity of illness was determined using SAPS II and SOFA. Clinical data (cardiovascular, respiratory, infective and metabolic parameters) were recorded at ICU admission and after 24 hours. Regarding renal function, we collected the pre-morbid SCr when available, SCr within 10 days of ICU admission, and SCr at discharge from the ICU. The glomerular filtration rate was estimated using the MDRD^34,35^ formula (eGFR) for the pre-morbid eGFR and on admission. All data was collected from the hospital electronic records and stored in a data set protected anonymously.

### Laboratory data

Blood and urine samples were immediately collected at ICU admission. Urinary [TIMP-2]∙[IGFBP7] was analysed with the NephroCheck® Test (Astute Medical, San Diego CA, USA). All values for [TIMP-2]*[IGFBP7] are reported in units of (ng/ml)^2^/1000. Serum creatinine was measured using the enzymatic method (IL testTM, Instrumentation® Laboratory SpA, Milan, Italy) on an ILab650 analyser (Instrumentation Laboratory, Werfen Group, Barcelona, Spain). All laboratory data was analysed by technicians who were blinded to the clinical data.

### Endpoints and statistical analysis

The primary endpoint was to determine the receiver operating characteristic curve (ROC) of the [TIMP-2]∙[IGFBP7] value at ICU admission to predict AKI within 24, 48 hours and 7 days. The secondary endpoints were: (a) to evaluate the ROC curve of the [TIMP-2]∙[IGFBP7] value at ICU admission to predict severe AKI (stages 2 and 3); (b) to identify the factors associated with AKI; and (c) to evaluate predictors of continuous renal replacement therapy (CRRT).

Quantitative variables were expressed by means and standard deviations and median (25th-75th) analysed by an independent Student’s t-test or the Welch-Satterthwaite correction for t-test in the event of an unequal variance or U- Mann Whitney test. Qualitative variables were reported by absolute frequencies and percentages and analysed by the Chi-Square Test or Fisher’s Exact Test, as appropriate. Normality was assessed using the Shapiro–Wilk test. A receiver operating characteristic curve (ROC) was created for [TIMP-2]*[IGFBP7]. Using the Liu method^15^, we determined an optimal cut-off and analyzed its sensitivity and specificity as well as the relative risk of AKI. To identify the predictors of AKI and CRRT, we performed a multivariable logistic regression, as appropriate. In the final model we included all predictors that were clinical or statistically significant in the univariate analysis to obtain the best model in terms of goodness of fit. In the case of collinearity, the variable with a “stronger” association (based on a forward selection process) was used in multivariable analysis. To calculate the discriminatory power of each model, we estimated the areas under the curve (AUC, 95% CI) and to quantify the improvement given by the biomarker [TIMP-2]*[IGFBP-7] through IDI (integrated discrimination improvement). We also assessed the calibration of the models by applying the Hosmer-Lemeshow test. A p-value of less than 0.05 was considered significant. Analyses were conducted using the STATA version 12.1 statistical software (StataCorp, 2012).

### Ethics approval and consent to participate

Ethical Committee of San Bortolo Hospital of Vicenza approved this study (protocol number 03/17). The consent to participate is pursuant to Italian laws.

## Supplementary information

Supplementary Information

## Data Availability

The datasets used and/or analysed during the current study are available from the corresponding author upon reasonable request. The corresponding author had full access to all of the data in the study and final responsibility for the decision to submit the study and data for publication.
