# Supplementary material for: Routine Adoption of Urinary [IGFBP7]∙[TIMP-2] to Assess Acute Kidney Injury at Any Stage 12 hours After Intensive Care Unit Admission: a Prospective Cohort Study
Source: Sci Rep. 2019 Nov 11;9:16484. doi: 10.1038/s41598-019-52790-6 (PMC6848119; doi:10.1038/s41598-019-52790-6)
Supplement: Supplementary file 1 — Supplementary Information [file 41598_2019_52790_MOESM1_ESM.pdf]

Supplementary Information

Routine Adoption of Urinary [IGFBP7]•[TIMP-2] to Assess Acute Kidney Injury at Any Stage 12 hours After Intensive Care Unit Admission: a Prospective Cohort Study

Fiorenza Ferrari, Gregorio Romero-González, Lilia Rizo Topete, Mara Senzolo, Anna Lorenzin, Faeq Husain-Syed, Mariangela Valentina Puci, Ottavia Eleonora Ferraro, Eva Muraro, Mara Serrano-Soto, Alejandra Molano Triviño, Ana Coutinho Castro, Yun Xie, Bo Yang, Massimo De Cal, Valentina Corradi, Alessandra Brendolan, Marta Scarpa, Maria Rosa Carta, Davide Giavarina, Raffaele Bonato, and Claudio Ronco.

Table 1S. AKI stage occurrence based on [TIMP-2]•[IGFBP7] cut-off [1].

AKI_stage	[TIMP-2]•[IGFBP7]≤ 0.3	[TIMP-2]•[IGFBP7]>0.3	Total
**Stage 1**	48	77	125
	80.00 %	60.16 %	66.49 %
**Stage 2**	6	28	34
	10.00 %	21.88%	18.09 %
**Stage 3**	6	23	29
	10.00 %	17.97%	15.43 %
**Total**	60	128	188
	100.00	100.00	100.00

Table 2S. Comparison of AKI prediction ability of [TIMP-2]•[IGFBP7]. Reference variable is AKI at different timepoints. Classification variable is [TIMP-2]•[IGFBP7] values at ICU admission. *All AKI* (any stage or stage 2 and 3) within 7 days means all AKI events up to 7 days; *AKI within 48 hours* (any stage or stage 2 and 3) means events occurred between 12 hours and 48 hours. *AKI after 48 hours* means AKI events occurs between 48 hours and 7 days. Since few patients developed severe AKI after 48 hours, ROC curve for AKI stage 2 and 3 after 48 hours has not been performed

Reference variable	Area under ROC curve	IC95%%	Standard error	Correctly classified	Sensitivity	Specificity	PPV	NPV
**AKI any stage within 12 hours**	0.74	0.69 - 0.80	0.03	75.11%	11.67%	98.18%	70.00%	75.35%
**AKI any stage within 48 hours**	0.70	0.65 – 0.76	0.02	65.43%	18.97%	96.89%	80.49%	63.85%
**AKI any stage after 48 hours**	0.40	0.28 -0.52	0.06	95.78%	0	100.00%	-	95.78%
**All AKI any stage within 7 days**	0.68	0.63 – 0.73	0.02	63.33%	20.21%	95.72%	78.00%	61.50%
**AKI stage 2 and 3 within 12 hours**	0.82	0.70 - 0.88	0.03	87.78%	5.56%	98.99%	42.86%	88.49%
**AKI stage 2 and 3 within 48 hours**	0.73	0.68 - 0.78	0.03	73.42%	11.11%	98.11%	70.00%	73.58%
**All AKI stage 2 and 3 within 7 days**	0.74	0.68 - 0.81	0.03	73.42%	11.11%	98.11%	70.00%	73.58%

AKI = Acute Kidney Injury; 2 ; **IGFBP7**= insulin-like growth factor-binding protein; **PPV**= Positive Predictive Value**; NPV**=Negative Predictive value; **TIMP-2**= tissue inhibitor of metalloproteinases

Table 3S. Empirical cut point estimation by Liu method [2]. Reference variable is showed in the left column and Classification variable is [TIMP-2]•[IGFBP7] values at ICU admission. *All AKI* (any stage or stage 2 and 3) *within 7 days* means all AKI events up to 7 days; *AKI within 48 hours* (any stage or stage 2 and 3) means events occurred from 12 hours to 48 hours. Since few patients developed severe AKI after 48 hours, empirical optimal cut point for AKI stage 2 and 3 after 48 hours has not been performed.

Reference variable	Empirical optimal cut point $((ng/ml)^2/1000)$	Area under ROC curve at cut point	Sensitivity at cut point	Specificity at cut point
**AKI any stage within 12 hours**	0.57	0.70	0.69	0.72
**AKI stage 2 and 3 within 12 hours**	1.7	0.76	0.67	0.84
**AKI any stage within 48 hours**	0.44	0.67	0.67	0.68
**AKI stage 2 and 3 within 48 hours**	0.45	0.70	0.73	0.66
**All AKI any stage within 7 days**	0.44	0.66	0.63	0.68
**All AKI stage 2 and 3 within 7 days**	0.58	0.68	0.70	0.67

Table 4S. Details on sensitivity/specificity for cut point described by Hoste, E.A.J et al. [1] in our study population. *All AKI* (any stage or stage 2 and 3) *within 7 days* means all AKI events up to 7 days; *AKI within 48 hours* (any stage or stage 2 and 3) means events occurred from 12 hours to 48 hours. Since few patients developed severe AKI after 48 hours, Hoste's cut point for AKI stage 2 and 3 after 48 hours has not been performed.

Reference variable	Hoste's cut point $(((ng/ml)^2/1000)$	Sensitivity at cut point > 0.3 $((ng/ml)^2/1000)$	Specificity at cut point >0.3 $((ng/ml)^2/1000)$
**AKI any stage within 12 hours**	> 0.3	0.80	0.54
**AKI stage 2 and 3 within 12 hours**	> 0.3	0.91	0.5
**AKI any stage within 48 hours**	> 0.3	0.72	0.56
**AKI stage 2 and 3 within 48 hours**	> 0.3	0.78	0.54
**All AKI any stage within 7 days**	> 0.3	0.67	0.56
**All AKI stage 2 and 3 within 7 days**	> 0.3	0.82	0.5

Figure 1S. The flow diagram shows patient recruitment, enrolment and AKI occurrence. From June 1 to December 31, 2016, 450 patients were admitted to the ICU at San Bortolo Hospital, Vicenza (Italy), 8 of whom did not meet the inclusion criteria. Serum creatinine was measured in all patients for 10 consecutive days and at ICU discharge. [TIMP-2]*[IGFBP7]* was measured at ICU admission.

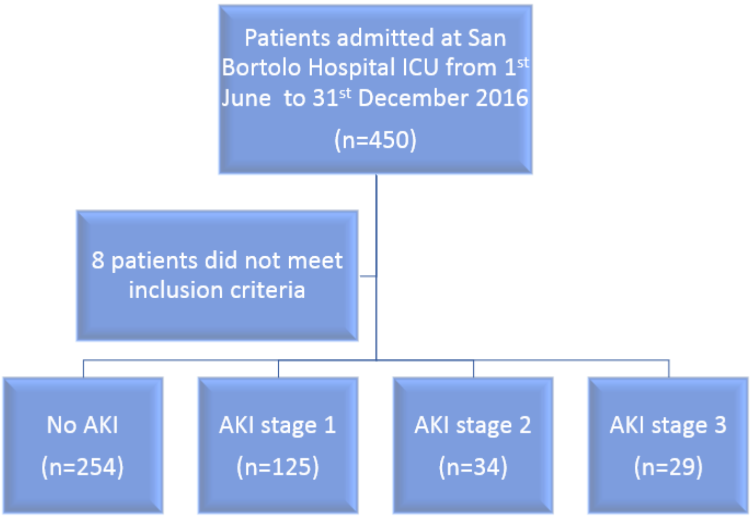

References

1. Hoste, E.A.J., McCullough, P.A., Kashani, K. et al. Derivation and validation of cutoff for clinical use of cycle arrest biomarkers. Nephrol Dial Transplant 29,2054-2061 (2014).
2. Liu, X. “Classification accuracy and cut point selection,” Stat Med. 31, 2676-86 (2012) doi: 10.1002/sim.4509.
